# Efficacy of electro-acupuncture on pregnancy outcomes for women undergoing *in vitro* fertilization: study protocol for a pilot randomized controlled trial

**DOI:** 10.3389/fendo.2024.1380885

**Published:** 2024-08-05

**Authors:** Xiaoyan Zheng, Ran Ji, Chaoliang Li, Hao Zhu, Ziyi Jiang, Jingwen Zhang, Yang Su, Siyi Yu, Zheng Yu, Xingyu Lv, Fanrong Liang, Jie Yang

**Affiliations:** ^1^ Acupuncture and Tuina School, Chengdu University of Traditional Chinese Medicine, Chengdu, China; ^2^ Clinical Research Center for Acupuncture and Moxibustion in Sichuan Province, Sichuan Jinxin Xi’nan Women and Children Hospital, Chengdu, China; ^3^ Intelligent Medicine School, Chengdu University of Traditional Chinese Medicine, Chengdu, China; ^4^ The Reproductive Center, Sichuan Jinxin Xi’nan Women and Children Hospital, Chengdu, China

**Keywords:** electro-acupuncture, *in vitro* fertilization (IVF), clinical pregnancy rate (CPR), pilot trial, randomized controlled trial (RCT), protocol

## Abstract

**Introduction:**

*In vitro* fertilization (IVF) is a technology that assists couples experiencing infertility to conceive children. However, unsuccessful attempts can lead to significant physical and financial strain. Some individuals opt for electro-acupuncture (EA) during IVF, even though there is limited evidence regarding the efficacy of this practice. Thus, this pilot study aims to explore the effectiveness and safety of EA during IVF on pregnancy outcomes.

**Methods and analysis:**

This clinical trial is a parallel, randomized, sham-controlled study. It aims to include a total of 118 infertile women who intend to undergo IVF. The participants will be randomly divided into three groups in a 1:1:1 ratio: the EA + IVF group, the placebo electro-acupuncture (pEA) +IVF group, and the IVF control group. All of the patients will be required to use ovarian stimulation drugs, while those in the EA + IVF and pEA + IVF groups will receive acupuncture treatment at three sessions per week (every other day) until trigger day with a minimum five session. The primary outcome of this trial will focus on the clinical pregnancy rate (CPR). CPR is defined as the rate of achieving clinical pregnancy from the first fresh/frozen embryo transfer cycle with an ultrasound-confirmed gestational sac in the uterine cavity. The secondary outcomes will assess embryology data, biochemical pregnancy rate, early miscarriage rate, Self-rating Anxiety Scale (SAS), Self-rating Depression Scale (SDS), Pittsburgh Sleep Quality Index (PSQI), Fertile Quality of Life (FertiQoL), patient retention rate, treatment adherence, and safety outcomes.

**Ethics and dissemination:**

Ethics approval was obtained from the Ethics Committee of Sichuan Jinxin Xi’nan Women and Children Hospital (number 2021–007). The results will be disseminated through peer-reviewed publications. The participants gave informed consent to participate in the study before taking part in it.

**Clinical trial registration:**

https://www.chictr.org.cn, identifier ChiCTR2300074455.

## Highlights

- This study is the first to provide information about the effectiveness and safety of electro-acupuncture (EA) treatment for infertile women undergoing *in vitro* fertilization (IVF). The study compares EA *versus* placebo EA and a control group.
- The study will use a rigorous methodology to reduce the risk of bias, including adequate randomization, quality control, and blinded outcome assessors and statisticians. Eligible participants will be strictly recruited and screened, and a standard operating procedure will be conducted rigorously to ensure quality control.
- Due to the nature of acupuncture, only EA and pEA group participants can be blinded. Treatment providers are likely to bring bias and influence the results.

## Introduction

Infertility is a global health issue affecting millions of people of reproductive age worldwide; it is defined as the failure to achieve a successful pregnancy after ≥12 months of regular, unprotected sexual intercourse (1). People with infertility are estimated to be at 48 to 186 million worldwide (2). Approximately 2.0% of all infants born in the United States each year are conceived by assisted reproductive technology (ART) (3). Despite technological advances, the average live birth rate by *in vitro* fertilization (IVF) remains low, with only 23%–26.9% per embryo transferred (4–6). The financial burden of repeated IVF cycles can be substantial, placing a significant economic pressure on affected patients and their families. Therefore, ensuring that the procedure’s efficiency is maximized while providing effective and safe strategies to assist infertile couples is imperative.

Patients have turned to complementary and alternative medical (CAM) treatments to increase the success rate of IVF. Among these CAM treatments, acupuncture is a frequently used adjunctive therapy (7). Since the first RCT reported by Paulus (8) in 2002 suggesting that acupuncture can increase the clinical pregnancy rate (CPR) of IVF, the application of acupuncture to IVF has attracted great interest from the international community. However, previous randomized clinical trials (RCT) (9–11) prevented a thorough evaluation of the beneficial effects of acupuncture in improving pregnancy outcomes with statistical heterogeneity (10); therefore, the efficacy is still controversial and remains debatable.

Acupuncture has a dose–effect relationship. Although patients may benefit from two acupuncture treatment sessions (8), many studies (12–14) failed to repeat this outcome. In our previous meta-analysis (15), a clear dose–response relationship with the pregnancy outcomes was revealed. Another study (16) suggested that patients receiving acupuncture before the stimulation of IVF, at two sessions per week for more than 4 weeks, may benefit more than those patients treated just pre-/post-ET. Based on previous clinical experience, we speculate that three sessions per week until the human chorionic gonadotropin (hCG) trigger day may be an effective therapy for women undergoing IVF.

Therefore, we have proposed to conduct a prospective, parallel, randomized, placebo-controlled trial that will be carried out to investigate the efficacy and safety of acupuncture treatment for patients with infertility, comparing them with placebo electro-acupuncture and IVF control groups. The current pilot study aims to assess the feasibility of performing a large-scale RCT, including patient recruitment, retention, and treatment adherence.

## Methods and design

### Study design

**Figure 1:**
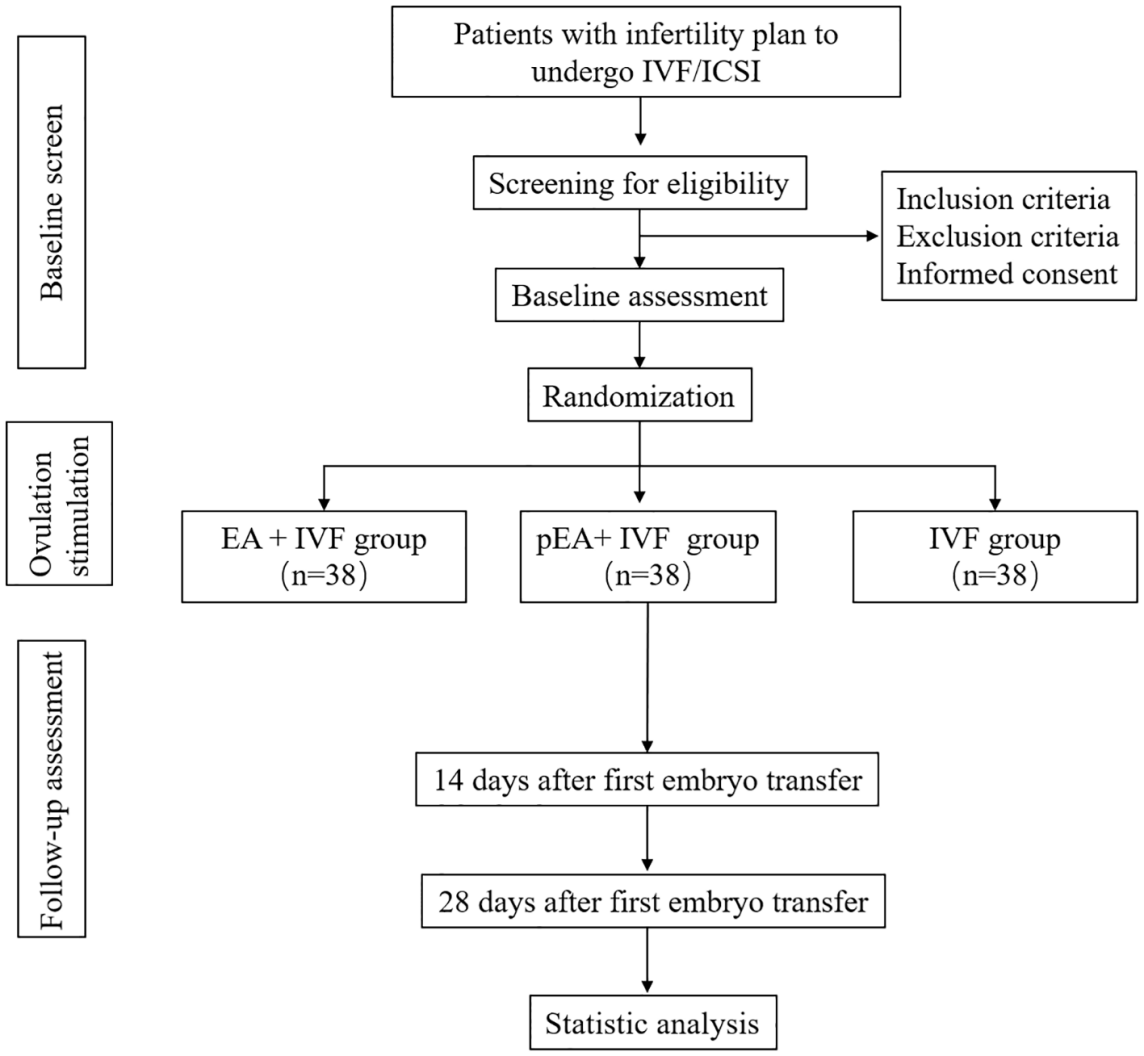
Flow diagram. IVF, *in vitro* fertilization; EA, electro-acupuncture.

This randomized, sham-controlled, prospective randomized trial will be conducted at the reproductive center of ichuan Jinxin Xi’nan Women and Children Hospital. The protocol has received approval from the Ethics Committee of Sichuan Jinxin Xi’nan Women and Children Hospital (number 2021–007) and has been registered at ClinicalTrials.gov (ChiCTR2300074455). The protocol will report with the Standard Protocol Items: Recommendations for Interventional Trials (SPIRIT) guidelines (17) and Standards for Reporting Interventions in Controlled Trials of Acupuncture (STRICTA) (18). The placebo acupuncture will be reported with Sham Acupuncture Reporting (SHARE) guidelines (19). **Figure 1** displays a flow chart of the trial. In addition, the schedule of enrollment, interventions, and assessments during the study period is shown in **Table 1**.

**Table 1:** Schedule of recruitment, intervention, and measures.

Items	Enrolment	Intervention	Follow-up			
	Screening and baseline assessment	Controlled ovarian hyperstimulation	Oocyte retrieval	Embryo transfer	14 days after embryo transfer	28 days after embryo transfer
Enrolment						
Eligibility screen	×					
Informed consent	×					
Allocation	×					
Baseline data	×					
Randomization						
Randomization	×					
Allocation	×					
Interventions						
EA/ pEA treatment		×				
COH treatment		×				
Assessments						
Laboratory tests	×	×	×	×	×	×
Embryo quality				×		
Pregnancy tests					×	×
Pregnancy outcomes				×	×	×
Fetus information						
FertiQoL	×		×			
SAS	×		×			
SDS	×		×			
PSQI	×		×			
Blinding assessments			×			
Adverse events		×	×	×	×	×
Retention rate						×
Adherence to treatment						×

EA, electro-acupuncture; pEA, placebo electro-acupuncture; COH, controlled ovarian hyperstimulation; FertiQoL, fertile quality of life; SAS, self-rating anxiety scale; SDS, self-rating depression scale. PSQI, Pittsburgh Sleep Quality Index.

### Recruitment of participants

Women diagnosed with infertility and undergoing IVF will be recruited through advertisements on the official accounts of Sichuan Jinxin Xi’nan Women and Children Hospital and through posters and WeChat. The clinical doctors of the reproductive centers will make the diagnoses based on the chief complaints, present illness, and physical examinations (1). The patients will be well-informed and write the consent before randomization.

#### Inclusion criteria

Women who are diagnosed with infertility (1) and will undergo IVF or Intracytoplasmic sperm injection (ICSI) who have met the following criteria will be recruited: (1) ages between 21 to 42 years old, (2) with GnRH-a or GnRH-A ovarian stimulation protocol, (3) AMH >1.2 ng/mL (20), (4) previous IVF/ICSI cycle ≤2, and (5) written informed consent.

#### Exclusion criteria

Patients will be excluded due to (1) preimplantation genetic testing (PGT), (2) malformation of the uterus: unicornuate uterus, bicornuate uterus, bicornuate uterus, or untreated uterine septum, (3) damage to the endometrial cavity or not cured diseases, such as submucosal myoma and uterine adenomyosis uterine adhesions or scar uterus, (4) diagnosed with an endocrine disorder: untreated hyperprolactinemia, hypothyroidism, hyperthyroidism, hyperandrogenism, chronic adrenocortical, and hypofunction, (5) diagnosed with polycystic ovary syndrome (PCOS), (6) having received acupuncture for infertility within the past 3 months, and (7) with bleeding tendency and infection, or severe allergies, or skin ulcers; scars are prohibited from acupuncture.

### Randomization and allocation concealment

Randomization will be conducted after the patients agree to consent to undertaking acupuncture treatments. Patients will be randomized according to the randomized number table generated by SPSS (version 27.0, IBM, Armonk, NY, USA) after completing the baseline assessments. Patients will be randomized into three groups: electro-acupuncture with IVF treatment (EA+ IVF group), placebo electro-acupuncture with IVF treatment (pEA + IVF group), and only IVF control (IVF group) in a 1:1:1 ratio using stratified block randomization (block = 3 or 6, randomly). An independent statistician with no clinical role in this study will comply with the randomization sequence. Allocation concealment was ensured by using a telephone by an independent third party to ensure objectivity and minimize the risk of bias.

### Blinding

Owing to the characteristics of the acupuncture procedure, the acupuncturists involved will not be blinded. In the EA + IVF group and pEA + IVF group, the single-blind will be conducted. The atmosphere of treatment, the communication between the acupuncturists and the patients, and the superficial appearance of the acupuncture techniques were the same. The apparatuses of the two groups are the same and can work normally. The study participants, fertility specialists, nurses, and the analyst were blinded to group allocation. The acupuncturists were not blinded to group allocation. Study site research physicians who were blinded to group allocation enrolled women in the study and collected clinical outcome data. Blinding was maintained until the completion of the analysis.

### Intervention

Patients in the three groups will receive GnRH-a or GnRH-A protocol according to the previous study (21, 22). Egg retrieval, fertilization, and embryo transfer were determined by their fertility specialists. During the study period, the patients’ medications, including dose and types, should be recorded in detail.

Following the randomization, the patients who were randomized into the EA + IVF group and the pEA + IVF group will receive EA treatment during the ovarian stimulation phase at three times per week (every other day) until trigger day with a minimum five session. Each session will continue for 30 min. Licensed acupuncturists possessing a minimum of 1 to 3 years of clinical experience will undergo training in a standardized operating procedure. This training aims to impart a comprehensive understanding of the precise locations of acupoints and non-acupoints. Additionally, they will acquire proficiency in needle manipulation and the operation of electro-devices. Only acupuncturists who have completed this training will be authorized to perform acupuncture treatments.

The disposable needles (Hwato, Suzhou, Jiangsu, China) and the SDZ-V EA apparatus (Suzhou Medical Appliance) have the same appearance. The needle device used for blinding is comprised of a sticky plastic plate with a guide tube. The needles and blunt-tip needles will be administered through the guide tube and the hole of the plastic plate.

#### Electro-acupuncture

**Figure 2:**
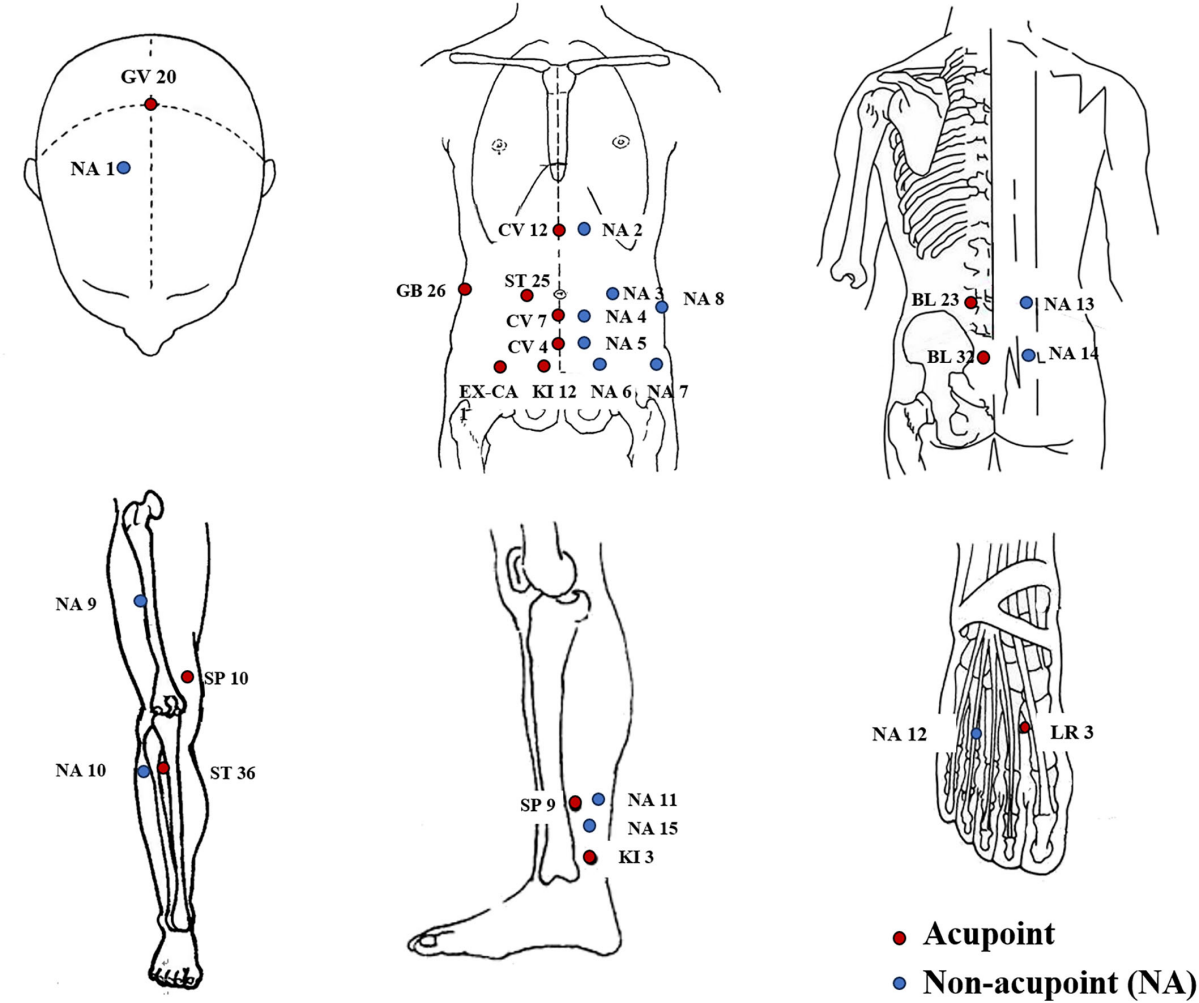
Locations of acupoints and non-acupoints. NA, non-acupoint.

The treatment protocol, guided by the TCM theory, is aimed to enhance ovarian response and endometrium receptivity and served as an adjunctive non-pharmacological therapy. The treatment will involve the alternation between two sets of acupuncture points. Set 1 will administer in the supine position, including GV20 (*Baihui*), CV12 (*Zhongwan*), ST25 (*Tianshu*), CV 6 (*Qihai*), CV4 (*Guanyuan*), GB26 (*Daimai*), KI12 (*Dahe*), EX-CA1 (*Zigong*), SP10 (*Xuehai*), ST36 (*Zusanli*), SP6 (*Sanyinjiao*), and LR3 (*Taichong*). Set 2 will administer in the prone position, including BL23 (*Shenshu*), BL32 (*Ciliao*), and KI3 (*Taixi*) (see details in **Table 2**, **Figure 2**). All of the acupoints will be located according to the “WHO Standard Acupuncture Point Locations in the Western Pacific Region” (23). Following needle insertion, small, equal manipulations of twirling, lifting, and thrusting will be performed on all needles to reach *De qi* (a composite of sensations including soreness, numbness, distention, heaviness, and other sensations) (18). Paired alligator clips from the EA apparatus will be attached transversely to the needle holders at bilateral ST25 and EX-CA1 and at bilateral ST36 and SP10 in the supine position, while needles at bilateral BL23 and BL32 were in the prone position. The EA stimulation will be with a dilatational wave of 2/100 Hz and a current intensity of 0.1 to 5 mA depending on the participant’s comfort level (preferably with skin around the acupoints shivering mildly without pain).

**Table 2:** Location of acupoints and manipulation techniques used for the EA+IVF group.

Acupoints	Location	Manipulation
Set 1 acupoints		
GV 20 (*Baihui*)	5 cun directly above the midpoint of the front hairline	Transverse insertion to a depth of 0.5 cun^a^–0.8 cun
CV12 (*Zhongwan)*	4 cun to the navel, on the upper abdomen middle	Inserted vertically to a depth of 1.0 cun–1.5 cun
ST25 (*Tianshu)*	Bilateral, 2 cun lateral to the umbilicus	Inserted vertically to a depth of 1.0 cun–1.5 cun
GB26 (D*aimai)*	Bilateral, 1.8 cun below to *Zhangmen* (*Zhangmen*, below the free end of the 11th floating rib)	Inserted vertically to a depth of 1.0 cun–1.5 cun
CV 6 (*Qihai*)	1.5 cun below the umbilicus, on the anterior midline	Inserted vertically to a depth of 1.0 cun–2.0 cun
CV4 (*Guanyuan*)	3 cun below the umbilicus, on the anterior midline	Inserted vertically to a depth of 1.0 cun–2.0 cun
KI12 (*Dahe)*	Bilateral, 4 cun below the umbilicus, and 0.5 cun lateral to the lower anterior midline	Inserted vertically to a depth of 0.5 cun–1.0 cun
EX-CA1 (*Zigong)*	Bilateral, 3 cun lateral to the umbilicus, and 4 cun lateral to the lower anterior midline	Oblique insertion to the uterus location direction to a depth of 0.8 cun–1.2 cun
SP10 (*Xuehai*)	Bilateral, 2 cun above the upper border of the patella	Inserted vertically to a depth of 1.0 cun–1.5 cun
ST36 (*Zusanli*)	Bilateral, 3 cun directly below *Dubi*, and one finger-breadth lateral to the anterior border of the tibia (*Dubi*, in the lateral depression of the patellar ligament, when the knee is flexed)	Inserted vertically to a depth of 1.0 cun– 2.0 cun
SP6 (*Sanyinjiao*)	Bilateral, 3 cun above the tip of the medial malleolus	Inserted vertically to a depth of 1.0 cun–1.5 cun
LR3 (*Taichong*)	Bilateral, in the depression anterior to the junction of the first and second metatarsal bones	Inserted vertically to a depth of 0.5 cun– 0.8 cun
Set 2 acupoints		
BL23 (*Shenshu*)	Bilateral, 1.5 cun lateral to the depression below the spinous process of the second lumbar vertebra	Inserted vertically to a depth of 0.5 cun–1.0 cun
BL 32 (*Ciliao*)	Bilateral, in the sacral region, below the anterior superior iliac spine, and precisely at the site of the second sacral foramen	Inserted vertically to a depth of 1.0 cun–1.5 cun
KI 3 (*Taixi*)	Bilateral, on the medial side of the foot, at the depression between the tip of the medial malleolus and the Achilles tendon	Inserted vertically to a depth of 0.5 cun–1.0 cun

a 1 cun (approximately 20 mm) is defined as the width of the interphalangeal joint of the patient’s thumb.

EA, electro-acupuncture; COH, controlled ovarian hyperstimulation.

#### Placebo electro-acupuncture

The participants in the pEA group will receive acupuncture with a pragmatic placebo needle on non-acupoints with no skin penetration. *Deqi* and manual stimulation in the group should be avoided. Non-acupoints (NA) deviate from the conventional acupoints or meridians. The locations of the non-acupoints are shown in **Table 3** and **Figure 2**. The same electrical stimulation device with a wave of 2/100 Hz and a current intensity of 0.1 mA will be applied. The paired electrodes will be connected to theneedles at bilateral NA-3 with NA-7 and at NA-9 with NA-10 in the supine position, while needles at bilateral NA-13 and NA-14 in the prone position will minimize electricity superficially.

**Table 3:** Location of points and manipulation techniques used for pEA+IVF study groups.

Acupoints	Location	Manipulation
Set 1 acupoints		
Non-acupoint 1	3.5 cun directly above the midpoint of the front hairline, 1 cun^a^ to GV21 (*Qianding*) (applied on body side alternatively on each treatment session)	Non-penetrating
Non-acupoint 2	4 cun above the umbilicus, 1 cun lateral to CV 12 (*Zhongwan*) (applied on body side alternatively on each treatment session)	Non-penetrating
Non-acupoint 3	Bilateral, 1 cun lateral to ST 25 (*Tianshu*), the midpoint between ST25 and SP15 (*Daheng*)	Non-penetrating
Non-acupoint 4	1.5 cun below the umbilicus, 1 cun lateral to CV6 (*Qihai*) (applied on body side alternatively on each treatment session)	Non-penetrating
Non-acupoint 5	3 cun below the umbilicus, 1 cun lateral to CV4 (*Guanyuan*) (applied on body side alternatively on each treatment session)	Non-penetrating
Non-acupoint 6	Bilateral, 0.5 cun lateral to KI 12 (*Dahe*), the midpoint between KI 12 and ST 29 (*Guilai*)	Non-penetrating
Non-acupoint 7	Bilateral, 0.5 cun lateral to EX-CA1 (*Zigong*), the midpoint between EX-CA1 and SP13 (*Fushe*)	Non-penetrating
Non-acupoint 8	Bilateral, 1 cun below to GB26 (D*aimai*)	Non-penetrating
Non-acupoint 9	Bilateral, 0.8 cun medial to the midpoint of the line linking the anterior superior iliac spine and the lateral end of the base of patella	Non-penetrating
Non-acupoint 10	Bilateral, 1 cun lateral to ST36 (*Zusanli*), between stomach meridian of foot Yangming and gallbladder meridian of foot Shaoyang	Non-penetrating
Non-acupoint 11	Bilateral, 3 cun above the tip of the medial malleolus between SP 6 (*Sanyinjiao*) and Achilles tendon	Non-penetrating
Non-acupoint 12	Bilateral, in the depression anterior to the junction of the third and fourth metatarsal bones	Non-penetrating
Set 2 acupoints		
Non-acupoint 13	Bilateral, 1 cun lateral to BL23 (*Shenshu*)	Non-penetrating
Non-acupoint 14	Bilateral, 1 cun lateral to BL32 (*Ciliao*)	Non-penetrating
Non-acupoint 15	Bilateral, 2 cun above KI 3(*Taixi*).	Non-penetrating

a 1 cun (approximately 20 mm) is defined as the width of the interphalangeal joint of the patient’s thumb.

EA, electro-acupuncture; COH, controlled ovarian hyperstimulation.

Throughout the intervention, the acupuncturists will maintain minimal verbal communication with the participants. Patients will be treated separately to prevent communication, and they were asked to document any other treatments that they had.

#### IVF control group

Patients in the IVF control group will not receive any EA treatment during the ovarian stimulation phase. After the end of the follow-up period, the participants will receive compensation after the follow-up period ends.

### Outcome measurement

The assessors will record the baseline characteristics, including the couple’s age, ethnicity, body mass index (BMI), diagnosis of infertility, infertility history, previous IVF cycles, the level of AMH, AFC, the level of FSH, and LH. Ovarian response parameters (duration of stimulation, total dose of gonadotrophins, peak E2 level, and number of oocytes retrieved) will be used to assess the ovarian function during the ovarian stimulation (**Table 4**). The duration of stimulation is calculated from the first day of administrating gonadotropin to the hCG trigger. After the treatment, the participants will be questioned about the group allocation that they received.

**Table 4:** Diagnosis definitions and time frame of outcomes.

Diagnosis	Definition	Time frame
Primary outcomes		
Clinical pregnancy^a^	Ultrasound-confirmed gestational sac	28 days after embryo transfer
Secondary outcomes		
Recruitment process		
Patient retention rate	The percentage of patients enrolled at baseline, who completed all follow-up measurements	Study completed
Adherence to treatment	Attendance at the treatment sessions	Study completed
Data on ovary induction		
Total dose of gonadotrophins (IU)	Was measured as the total dosage of gonadotropin drugs during the ovarian stimulation phase	On trigger day
Endometrium thickness (cm)	Was measured in the midsagittal plane of the thickness of the bilateral endometrium	On trigger day
Estrogen level pg/mL	Level of estrogen in the peripheral blood	On trigger day
Embryo data		
Ovum retrieved	Number of eggs retrieved from bilateral ovaries	On trigger day
High-quality embryos	Number of high quality measured by embryologists	3 or 5 days after the ovum was retrieved
Pregnancy outcomes		
Biochemical pregnancy^a^	Serum human chorionic gonadotropin ≥10 mIU/mL	10–14 days after embryo transfer
Early miscarriage^a^	Fetal growth stops or the absence of heart activity in the gestational sac within the first 12 weeks of pregnancy	Before 12 weeks in gestational age
Score of the questionnaires		
SAS	SAS consists of 20 items, and its measurement is determined by calculating the sum of the scores of each item	Baseline and on trigger day
SDS	SDS consists of 20 items, and its measurement is determined by calculating the sum of the scores of each item	Baseline and on trigger day
FertiQoL	FertiQoL contains 36 items, and its measurement is determined by calculating the sum of the scores of each item	Baseline and on trigger day
PSQI	PSQI contains 19 items, and its measurement is determined by calculating the sum of the scores of each item	Baseline and on trigger day
Adverse events		
	Including nausea during the ovarian stimulation, hematoma around the site of needling, infection around the site of needling, dizziness after acupuncture, and other discomforts after acupuncture	After each treatment

a The rates of pregnancy outcomes are defined as the number of pregnancy outcome cycle per ovarian stimulation cycle.

#### Primary outcome

In this feasibility and prior study, the primary outcome is the CPR, defined as the rate of achieving clinical pregnancy from the first fresh/frozen embryo transfer cycle with an ultrasound-confirmed gestational sac in the uterine cavity (24) as measured 28 days after embryo transfer.

#### Secondary outcomes

The secondary outcomes include embryology data, other pregnancy outcomes, and the scale and questionnaire measurement. The schedule of measurements and visits for each outcome are shown in **Table 4**. We will also observe the patient retention rate and treatment adherence. Retention rate is defined as the percentage of patients enrolled at baseline and who completed all follow-up measurements. Treatment adherence is assessed using attendance at the treatment sessions. Successful pilot targets are defined as the recruitment of 114 participants over 4 months, an 80% retention rate (25), and at least four treatment sessions attended.

Embryology data include total ova retrieved and total high-quality embryos. The high-quality embryos will be graded according to the guidelines (26, 27) by embryologists. Cleavage-stage embryos and blastocyst-stage embryos will be recorded separately.

The follow-up of pregnancy outcomes will be observed around 14 days and 12 weeks after embryo transfer. Besides CPR, the biochemical pregnancy rate (BPR), embryo implantation rate, and early miscarriage rate will also be observed, respectively. BPR is defined as the level of human chorionic gonadotropin (hCG) >5 mIU/mL (28). The fertilization rate is defined as the number of 2PN embryos divided by the number of inseminated oocytes. The early miscarriage rate is defined as the fetal growth stops or the absence of heart activity in the gestational sac within the first 12 weeks of pregnancy.

#### Scale and questionnaire measurement

Anxiety and depression are the most prevalent mental illnesses contributing to the global infertility burden, which are also potential risk factors for IVF success. Emotional disorder can also lead to sleep problems and will influence the quality of life. Therefore, the scale and questionnaire measurement will be measured at baseline and on the trigger day to learn about changes in the mental disorder after the acupuncture treatments (**Table 4**). Self-rating anxiety scale (SAS) (29) and the self-rating depression scale (SDS) (30) are 20-item Likert scales, with raw scores that range from 20 to 80, which are converted to index scores by dividing the sum of the raw scores by 80 and multiplying by 100. Higher scores indicate greater severity of anxiety or depression symptomology. Index scores, 25–49 (raw scores 20–40); normal, 50–59 (raw scores 41–47); mild to moderate, 60–69 (raw scores 48–55); moderate to severe; and 70 and over (raw scores 56 and over), severe (31). The Pittsburgh Sleep Quality Index (PSQI) is a self-rated questionnaire to assess sleep quality and disturbance that can be used in clinical practice and research studies for patients with psychiatric or sleep problems and even among the general population (32). It contains 19 items in seven subdomains, including subjective sleep quality, sleep latency, sleep duration, habitual sleep efficiency sleep disturbances, use of sleeping medication, and daytime dysfunction (32). The sum scores of each item range daytime dysfunction from 0 to 21. The higher the patients’ scores, the worse sleep quality they experience. The fertile quality of life (FertiQoL) is an instrument to measure the quality of life in men and women experiencing fertility problems (33). FertiQoL consists of 36 items that assess core (24 items) treatment-related quality of life (QoL) (10 items) and overall life and physical health (two items). The items in the prototype FertiQoL survey were randomly presented and rated on a scale of 0–4, where higher scores indicated a more favorable quality of life.

#### Blinding assessment

To test whether blinding is successful, patients in the EA + IVF group and pEA + IVF group will be asked to guess which kind of acupuncture they received at hCG trigger day after finishing the acupuncture treatment.

#### Safety outcomes

Adverse events (AE) will be monitored and promptly reported in this study through a dedicated safety monitoring team and a comprehensive reporting system. The team will track and document all adverse events, ensuring timely response and participant safety. AEs are described as dizziness, nausea, headache, or bruising. The causality with acupuncture will be analyzed by the acupuncturists. The occurrence, duration, severity (symptoms and signs), and corresponding solution of the adverse events should be recorded in detail in eCRFs. To ensure the safety of EA during the ovarian stimulation phase, all acupuncturists will be trained to perform EA according to standard operating procedures (SOPs). This ensures standardization and minimizes risks, guaranteeing the safety of participants during the IVF process.

### Data management

All researchers, including acupuncturists, outcome assessors, and statisticians, have received training in data management. The data will be entered into the CRF system, which was established before the recruitment process. The clinical research associates will be responsible for ensuring the accuracy of the data. Online monitoring will be used during the trial to ensure that the data is collected completely and accurately. The “check” function in the CRF system will assist with dynamic data management. Once the study is complete, the data management team will implement data lockup, and researchers will be unable to modify the data. All research documents, including paper files and electronic documents, will be kept for at least 5 years after publication. If reviewers or readers have any questions regarding the published data, they can contact the corresponding author for access to the original data. To ensure confidentiality, private patient information, such as names, telephone numbers, and identification numbers, will remain anonymous.

### Quality control

The trial protocol has been thoroughly reviewed and revised by experts in acupuncture, methodology, and statistics. Standard operating procedures have been established to conduct the intervention, complete the CRF, assess the outcomes, and manage data. The relevant staff has been trained accordingly to ensure adherence to these procedures. An inspection plan will be designed to ensure quality control. For patients, quality control can be carried out by randomly selecting several individuals and confirming their authenticity through phone calls. Patients and researchers will be spot-checked to ensure that their informed consent process is ethical. The key focus during the intervention will be to standardize the manipulation process to investigate whether the implementation process of acupuncture is carried out by this clinical study. Specifically, it includes whether the intervention implementer has received training for this study, the accuracy of the acupoint selection, the standardization of disinfection, the method and scale of needle insertion, and the standardization of manipulation. We will conduct face-to-face interviews with the intervention implementers, monitor the actual research process, or monitor the implementation process through simulation.

### Sample size

The purpose of this study was to evaluate the study’s feasibility and support the development of a future definitive RCT. Utilizing the upper confidence limit method, we determined that a pilot sample size of between 20 to 40 individuals can serve as a benchmark (34). Based on the consensus of the methodologists, statisticians, and acupuncturists, we decided that a sample size of 30 in each group (25) and a 20% estimated dropout rate were sufficient; thus, the total of 114 patients would be considerable (38 in each group).

### Statistical analysis

Intention-to-treat (ITT) analysis encompassing all randomized patients will be conducted for all efficacy analyses. Additionally, a per-protocol (PP) analysis focusing on the primary outcome will be performed as a sensitive analysis, excluding patients who did not complete the acupuncture treatment. Missing outcome data will be complemented using multiple imputations with the package “Mice” from the R software (www.r-project.org).

Categorical variables will be reported by number and percentage, whereas continuous variables will be represented by mean and standard deviation (SD) (or median and interquartile range, IQR). The retention rate and treatment adherence will be reported in descriptive statistics.

For the primary outcome, CPR will be compared by the proportions of included patients with clinical pregnancy. To determine the independent factors, logistic regression will be used to identify baseline variables that are associated with outcomes. The retention rate and rates of biochemical pregnancy, ongoing pregnancy, blinding assessments, and adverse events will undergo statistical analysis using chi-squared tests or Fisher’s exact test. Other outcomes will be evaluated using one-way variance (ANOVA) models or Kruskal–Wallis analysis. The score of SAS, SDS, FertiQol, and PSQI, respectively, will be assessed at baseline and after treatment among groups on hCG trigger day. Statistical significance will be determined at *P <*0.05. Pairwise comparisons will be performed between the EA + IVF group and the pEA + IVF group and between the EA + IVF group and the IVF group in a *post hoc* analysis.

A predetermined subgroup analysis will be performed to determine whether the baseline characteristics (female age, ovarian stimulation protocol, etc.) affect the CPR to EA. The data was analyzed using SPSS version 27.0 (IBM, Armonk, NY, USA). As this is a pilot study, our results were considered exploratory.

## Discussion

Recurrent IVF failure significantly impairs the patients’ quality of life and leads to a great economic burden for the families as well as society. This parallel, three-arm, randomized, placebo-controlled trial will assess the feasibility of acupuncture in treating infertile women who will undergo IVF.

This trial meets the methodological demand for adequate randomization, allocation concealment, and blinding of patients, outcome assessors, and statisticians. In this study design, there are several factors different from previous studies. Firstly, three treatment sessions per week during the ovarian stimulation and at least five sessions will be considerable. Previous studies conducted two before embryo transfer (8, 35), or one session was added on the 6–8 days of ovarian stimulation (three sessions in total) (9, 36). In these trials, the dose of acupuncture was far from adequate. In our previous meta-research, a high dosage of treatment showed better results for pregnancy outcomes. As a CAM, EA aims to improve the quality of the oocyte; enough dosage acupuncture will be needed as Magarelli (16) and White (37) have suggested. Secondly, EA with 2/100 Hz will be administered during the ovarian stimulation. In 1996, Stener-Victorin (38) found that EA can reduce the blood flow impedance in the uterine arteries. It is suggested that both of these effects are due to a central inhibition of the sympathetic activity. Moreover, Stener-Victorin reported that EA inhibits hyperactivity in the sympathetic nervous system in influencing ovarian morphology (39). For the tensity of electro-acupuncture, low tensity with 2–50 Hz was utilized frequently, especially in TEAS. However, low density and high tensity alternately will be more beneficial for long-duration treatment. Thus, this study is designed using EA with 2/100 Hz. Thirdly, acupoints were summarized from bibliographic retrieval. In total, 87 studies of acupuncture in treating infertility were screened and subjected to quality assessment. We ranked the acupoints with a high frequency utilized. CV12 (*Zhongwan*), ST25 (*Tianshu*), CV 6 (*Qihai*), CV4 (*Guanyuan*), KI12(*Dahe*), EX-CA1 (*Zigong*), SP10 (*Xuehai*), ST36 (*Zusanli*), SP6 (*Sanyinjiao*), LR3 (*Taichong*), BL23 (*Shenshu*), BL32 (*Ciliao*), and KI3 (*Taixi*) were recommended by 34 professors from all over the world. To our knowledge, CV 6 (*Qihai*), CV4 (*Guanyuan*), KI12 (*Dahe*), and EX-CA1 (*Zigong*) are positioned around nerves, which are connected with the ovaries and uterus (40). Blood vessels will be stimulated by nerve control. Moreover, the ovarian and uterus functions are governed by the hypothalamic–pituitary–ovarian (H–P–O) axis. Relaxing the depression and anxiety and improving the quality of life and sleep, the H–P–O axis might be influenced by stimulation. However, the placebo effect of EA cannot be ignored. We set a pEA in our pilot research; non-penetration of skin and blunt needles will be utilized to minimize the effect. To blind the patients successfully, we treated them separately and avoided communications.

There are certain limitations to consider. Firstly, due to the inherent nature of acupuncture, it is impractical to blind either the acupuncturists or the patients during the treatment process when comparing the EA + IVF group with the IVF control group. Secondly, our study was confined to a single-center trial involving 118 patients, potentially introducing selection bias. Nevertheless, for future trials, we aim to recruit patients from multiple centers in a significantly larger sample size to mitigate these limitations.

### Trial status

This study is currently in the recruitment phase. The first patient was randomized on August 15, 2023, and the study is expected to end in the middle of 2024.

## Ethics statement

The studies involving humans were approved by Sichuan Jinxin Xi’nan Women and Children Hospital. The studies were conducted in accordance with the local legislation and institutional requirements. The participants provided their written informed consent to participate in this study. Written informed consent was obtained from the individual(s) for the publication of any potentially identifiable images or data included in this article.

## Author contributions

XZ: Conceptualization, Formal analysis, Investigation, Methodology, Project administration, Supervision, Visualization, Writing – original draft, Writing – review & editing. RJ: Investigation, Writing – original draft. CL: Data curation, Investigation, Software, Supervision, Writing – original draft. HZ: Data curation, Investigation, Software, Writing – original draft. ZJ: Investigation, Writing – original draft. JZ: Investigation, Writing – original draft. YS: Investigation, Writing – original draft. SY: Formal analysis, Methodology, Writing – review & editing. ZY: Writing – review & editing, Investigation. XL: Conceptualization, Methodology, Writing – original draft. FL: Conceptualization, Writing – review & editing. JY: Conceptualization, Resources, Writing – review & editing.
